# APRIL is overexpressed in cancer: link with tumor progression

**DOI:** 10.1186/1471-2407-9-83

**Published:** 2009-03-16

**Authors:** Jérôme Moreaux, Jean-Luc Veyrune, John De Vos, Bernard Klein

**Affiliations:** 1 CHU Montpellier, Institute of Research in Biotherapy, Montpellier, France; 2 INSERM, U847, Montpellier, F-34197 France; 3 Université Montpellier1, UFR Médecine, Montpellier, France

## Abstract

**Background:**

BAFF and APRIL share two receptors – TACI and BCMA – and BAFF binds to a third receptor, BAFF-R. Increased expression of BAFF and APRIL is noted in hematological malignancies. BAFF and APRIL are essential for the survival of normal and malignant B lymphocytes, and altered expression of BAFF or APRIL or of their receptors (BCMA, TACI, or BAFF-R) have been reported in various B-cell malignancies including B-cell non-Hodgkin's lymphoma, chronic lymphocytic leukemia, Hodgkin's lymphoma, multiple myeloma, and Waldenstrom's macroglobulinemia.

**Methods:**

We compared the expression of *BAFF, APRIL, TACI and BAFF-R *gene expression in 40 human tumor types – brain, epithelial, lymphoid, germ cells – to that of their normal tissue counterparts using publicly available gene expression data, including the Oncomine Cancer Microarray database.

**Results:**

We found significant overexpression of *TACI *in multiple myeloma and thyroid carcinoma and an association between TACI expression and prognosis in lymphoma. Furthermore, *BAFF and APRIL *are overexpressed in many cancers and we show that *APRIL *expression is associated with tumor progression. We also found overexpression of at least one proteoglycan with heparan sulfate chains (HS), which are coreceptors for APRIL and TACI, in tumors where APRIL is either overexpressed or is a prognostic factor. APRIL could induce survival or proliferation directly through HS proteoglycans.

**Conclusion:**

Taken together, these data suggest that APRIL is a potential prognostic factor for a large array of malignancies.

## Background

APRIL and BAFF are two members of the TNF family. BAFF is a type II transmembrane protein that can be secreted after proteolytical cleavage from the cell membrane[1,2]. APRIL is processed intracellularly within the Golgi apparatus by a furin pro-protein convertase prior to secretion of the biologically active form[3]. APRIL can also be expressed as a cell surface fusion protein with TWEAK called TWE-PRIL[4,5]. Both ligands bind to TACI (transmembrane activator and CAML interactor) and BCMA (B-cell maturation antigen), two members of the TNFR family. BAFF binds additionally to BAFF receptor (BAFF-R). BAFF is involved in the survival of normal and malignant B cells and normal plasmablasts [6-8]. APRIL is highly expressed in several tumor tissues, stimulates the growth of tumor cells[9] and promotes survival of normal plasmablasts and plasma cells[10,11].

Evidence has been presented that BAFF/APRIL contribute to malignancies of B cells and plasma cells: non-Hodgkin's lymphoma [12-16], Hodgkin lymphoma[17], chronic lymphocytic leukemia[18,19], multiple myeloma [20-24] and Waldenstrom's macroglobulinemia[25].

Recombinant APRIL binds to several cell lines that do not express detectable mRNA for TACI and BCMA and proteoglycans were identified as APRIL-specific binding partners. This binding is mediated by heparan sulfate (HS) side chains and can be inhibited by heparin[26,27]. Binding of APRIL to proteoglycans or BCMA/TACI involves different regions in APRIL. APRIL binds HS proteoglycans via the lysine-rich region in the N-terminal part, leaving the TNF-like region available to interact with others receptors. Blockade of APRIL/BAFF using human BCMA-Ig in nude mice inhibited the growth of a subcutaneously injected human lung carcinoma cell line (A549) and a human colon carcinoma cell line (HT29)[28]. These cell lines express APRIL, but not BAFF, TACI, BCMA or BAFF-R suggesting that HS proteoglycans could mediate the growth response to APRIL. However, BCMA-Fc leaves the APRIL binding HSPG domain intact. This blockade may suggest that the TNF-receptor binding domain is also necessary for activity, and that an additional APRIL-specific receptor might exist. B-cell lymphoma cells can bind large amount of APRIL secreted by neutrophils via proteoglycan binding and the high expression of APRIL in tumor lesion correlates with B-cell lymphoma aggressiveness[16]. More recently, Bischof *et al *demonstrated that TACI binds also HS proteoglycans like syndecan-1, syndecan-2 and syndecan-4 [29].

These data demonstrate that BAFF/APRIL are potent growth factors in B cell malignancies. Furthermore, APRIL could be implicated in tumor emergence and/or progression due to its ability to bind HS proteoglycans [26-28]. Therefore, we looked for the expression of *BAFF, APRIL *and of their receptors – *BAFF-R, BCMA*, and *TACI *– in various cancers, compared to their normal tissue or cell counterparts and in association with disease staging.

## Methods

### Databases

We used Oncomine Cancer Microarray database http://www.oncomine.org[30] and Amazonia database http://amazonia.montp.inserm.fr/[31] to study gene expression of *BAFF, APRIL, BCMA, TACI, BAFF-R and HS proteoglycans *genes in 40 human tumor types and their normal tissue counterparts as indicated in Table 1 (Additional file 1). Only gene expression data obtained from a single study using the same methodology were compared. All data were log transformed, median centred per array, and the standard deviation was normalized to one per array[32].

### Statistical analysis

Statistical comparisons were done with Mann-Whitney or Student t-tests.

## Results and Discussion

### BAFF is overexpressed in solid tumors

**Figure 1:**
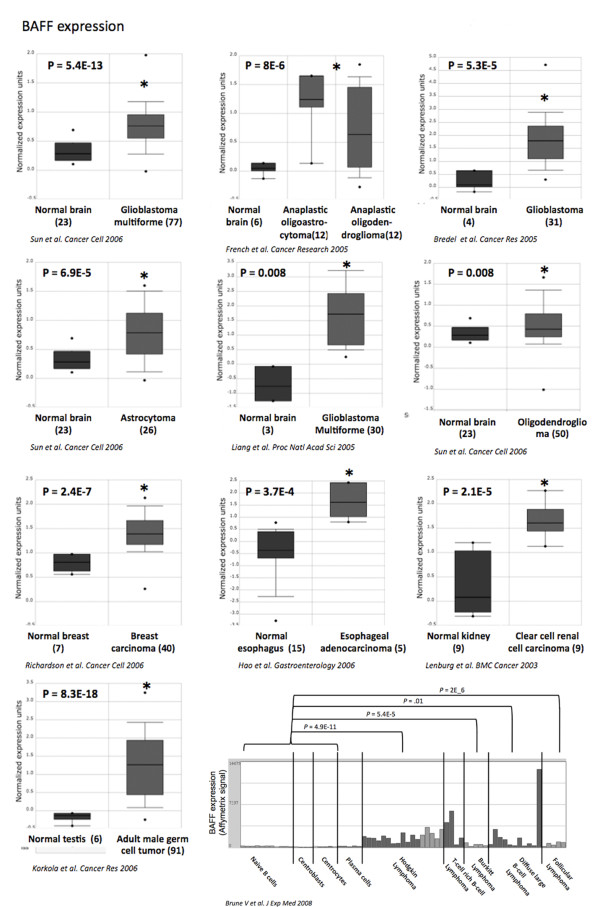
**BAFF expression in various cancers**. *BAFF *gene expression in normal brain, glioblastoma multiforme [34-37], normal breast, breast carcinoma[40], normal esophagus, esophageal adenocarcinoma[41], normal kidney, renal carcinoma[42], normal testis, adult male germ cell tumor[43], Hodgkin lymphoma, Burkitt lymphoma, diffuse large B-cell lymphoma and follicular lymphoma[33]. Data sets in a single panel were from the same study. GEP data are log transformed and normalized as previously described[32]. In brackets, are indicated the number of normal or tumor samples.

40 tumor types were investigated, including 36 solid tumors and 4 hematological tumors (Additional file 1: Table 1). BAFF gene is overexpressed in 1/4 hematological tumors and in 5/36 solid tumors. BAFF is overexpressed in Hodgkin lymhoma compared to normal B cells (*P *= 4.9E-11), in Burkitt lymphoma compared to normal B cells (*P *= 5.4E-5), in diffuse large B cell lymphoma compared to normal B cells (*P *= .01) and in follicular lymphoma compared to normal B cells (*P *= 2E-6)[33]. Four independent studies have shown *BAFF *overexpression in glioblastoma compared to normal brain (*P *= 5.4E-13, *P *= 8E-6, *P *= 5.3E-5 and *P *= 0.008) [34-37] (Figure 1). Following stimulation with inflammatory cytokines, astrocytes *in vitro *produce high amounts of BAFF. *BAFF *is expressed in astrocytes in the normal human central nervous system and is strongly upregulated in activated astrocytes in the demyelinated lesions of multiple sclerosis[38]. BAFF secretion could be relevant in sustaining intrathecal B cell responses in autoimmune and infectious diseases of the central nervous system. Accordingly, *BAFF *expression is readily induced in the central nervous system by neurotrophic viruses and correlates with the recruitment of antibody-secreting cells[39]. The overexpression of BAFF in brain tumors could be linked to the accumulation of tumor cells or to inflammatory signals. Regarding other cancers, *BAFF *was found significantly overexpressed in breast carcinoma compared to normal breast (Richardson et al[40]; *P *= 2.4E-7), in esophageal adenocarcinoma compared to normal esophagus (Hao et al[41]; *P *= 3.7E-4), in clear cell renal cell carcinoma compared to normal kidney (Lenburg et al[42]; *P *= 2.1E-5) and in adult male germ cell tumor compared to normal testis (Korkola et al[43]; *P *= 8.3E-18).

### APRIL is overexpressed in solid and hematological malignancies

**Figure 2:**
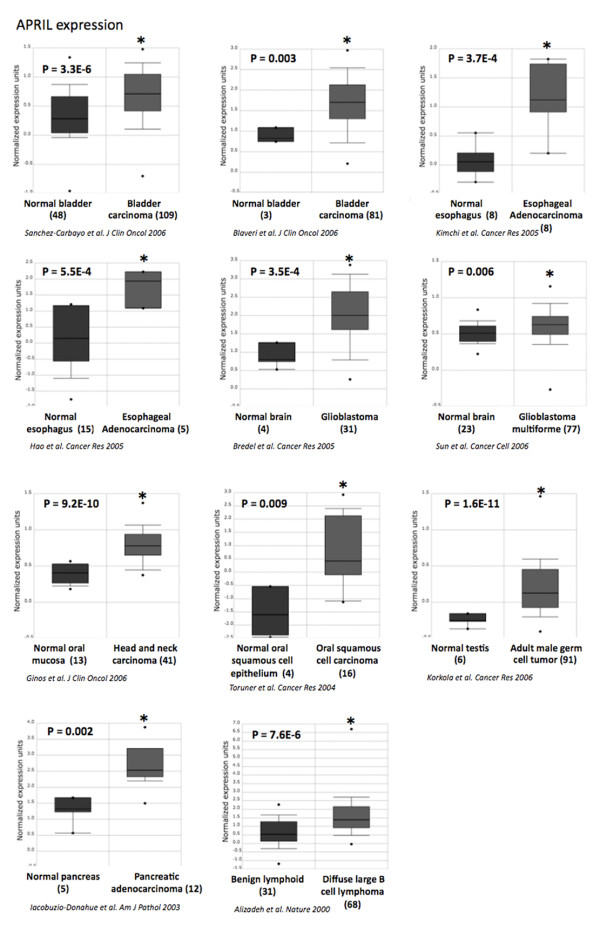
**APRIL expression in various cancers**. APRIL gene expression in normal bladder, bladder carcinoma[44,45], normal esophagus, esophageal adenocarcinoma[41,46], normal brain, glioblastoma multiform[34,36], normal oral mucosa, head and neck carcinoma[47,48], normal B cell, lymphoma[49,50], normal pancreas, pancreatic adenocarcinoma[51], normal testis and adult male germ cell tumor[43]. Data sets in a single panel were from the same study. GEP data are log transformed and normalized as previously described[32]. In brackets, are indicated the number of normal or tumor samples.

*APRIL *gene was overexpressed in 1/4 hematological and in 6/36 solid tumors in tumors (Additional file 1: Table 1). *APRIL *was overexpressed in invasive bladder carcinoma compared to superficial bladder carcinoma in two independent studies (*P *= 3.3E-6 and *P *= 0.003)[44,45], in esophageal adenocarcinoma compared to normal esophagus in two independent studies (*P *= 3.7E-4 and *P *= 5.5E-4)[41,46], in glioblastoma compared to normal brain in two independent studies (*P *= 3.5E-4 and *P *= 0.006)[34,36], in head and neck carcinoma compared to normal oral mucosa in two independent studies (*P *= 9.2E-10 and *P *= 0.009)[47,48], in diffuse large B cell lymphoma compared to normal lymph nodes (*P *= 7.6E-6)[49,50], in pancreatic adenocarcinoma compared to normal pancreas (Iocobuzio-Donahue et al[51]; *P *= 0.002) and in adult male germ cell tumor compared to normal testis (Korkola et al[43]; *P *= 1.6E-11) (Figure 2).

**Figure 3:**
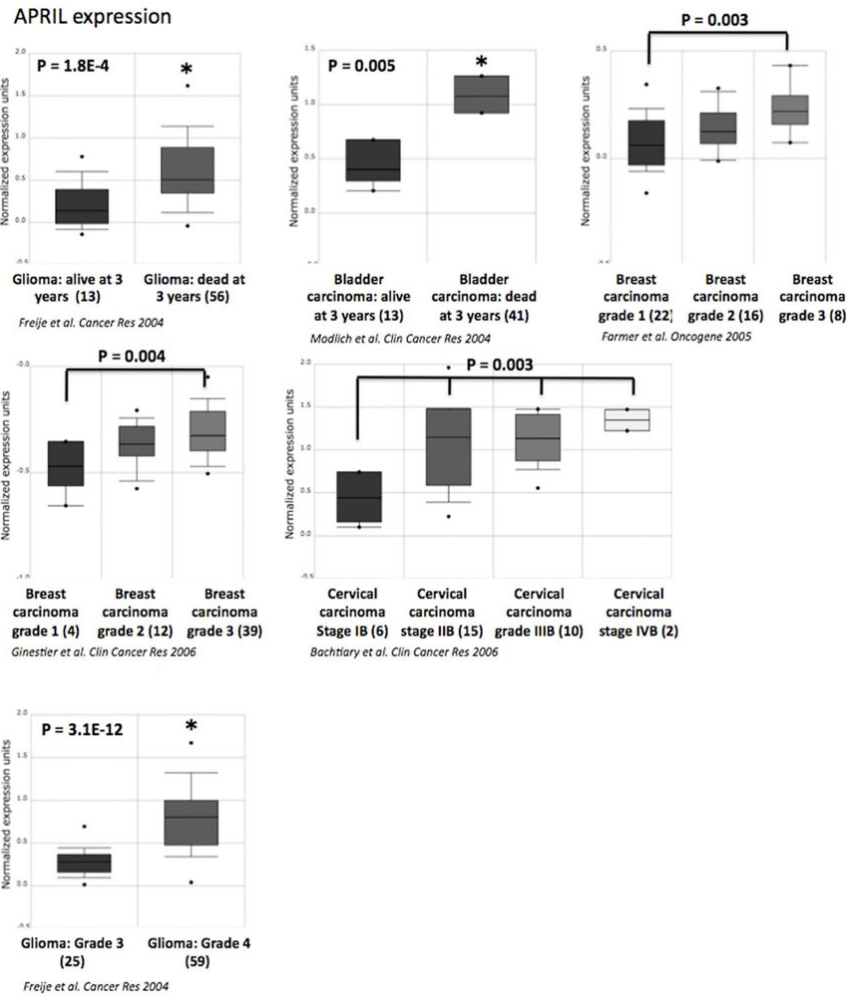
**Association between APRIL expression and aggressiveness in various cancers**. APRIL gene expression in glioma[52], bladder carcinoma[53], breast carcinoma[54,55] and cervical carcinoma[56]. Data sets in a single panel were from the same study. GEP data are log transformed and normalized as previously described[32]. In brackets, are indicated the number of normal or tumor samples.

Recently, Schwaller *et al*[16] demonstrated that high *APRIL *expression in tumor lesions correlates with B-cell lymphoma aggressiveness. We found here that *BAFF or APRIL *expression could be also associated with tumor aggressiveness in other cancers (Figure 3). *APRIL *is significantly overexpressed in glioma grade 4 compared to glioma grade 3 (*P *= 3.1E-12) and in patients presenting glioma, dead at 3 years compared to patients alive at 3 years (*P *= 1.8E-4)[52] (Additional file 2). In bladder carcinoma, *APRIL *is overexpressed in patients dead at 3 years compared to patients alive at 3 years (*P *= .005)[53]. High tumor cell mass in breast cancer was also characterized by increased *APRIL *expression in two independent studies (*P *= .003 and *P *= .004)[54,55]. Stages II, III and IV cervical carcinoma showed an overexpression of *APRIL *compared to stage I (*P *= 0.003)[56] (Figure 3). On the contrary, BAFF overexpression was not associated with prognosis or tumor aggressiveness using online cancer gene expression analysis[32]. APRIL protein was detected in several human solid tumors[57] and normal epithelial cells[11]. Interestingly, APRIL protein expression was reported in larynx and oral cavity carcinoma, esophagus carcinoma, bladder carcinoma, breast cancer, pancreatic tumors, ovarian carcinoma, seminoma adenocarcinoma, glioblastoma multiforme and oligodendroglioma correlating with the results of our analysis[57,58]. In addition, neutrophils, present in the stroma of solid tumors, are major producers of APRIL [57] suggesting that APRIL overexpression may be due to infiltrating cells. Furthermore, APRIL that is produced in tumors is retained and accumulates in the tumor stroma though HSPG binding[57]. Nevertheless, an upregulation of APRIL protein (detected by in situ immunostaining) was shown to not alter disease-free and overall survivals of bladder, ovarian and head and neck carcinoma patients in retrospective studies[58]. We have identified here a significant overexpression of *APRIL *in patients with glioma or bladder carcinomas who were dead at 3 years compared to patients alive at 3 years. An explanation could be that a part of APRIL protein is quickly secreted and no more detectable by immunostaining. APRIL protein could also be used by tumor cells as a growth factor or released in the blood circulation.

### TACI is overexpressed in hematological cancers

**Figure 4:**
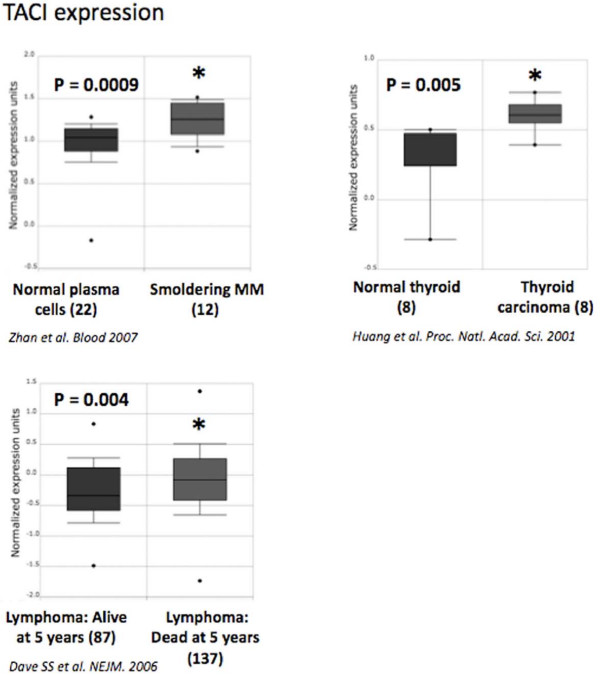
**TACI expression in various cancers**. *TACI *gene expression in normal plasma cells, smoldering myeloma[60], normal thyroid, thyroid carcinoma[61] and lymphoma[62]. Data sets in a single panel were from the same study. GEP data are log transformed and normalized as previously described[32]. In brackets, are indicated the number of normal or tumor samples.

We have previously reported the relevance of Affymetrix microarrays to quantify *BCMA *and *TACI *expressions in MM since microarray data were validated by real time RT-PCR and flow cytometry[20,21,59]. No differences in *BCMA *or *BAFF-R *expression between tumor cells and their normal counterparts could be found in the 40 cancer types available from Oncomine database. By contrast, a significant *TACI *overexpression was found in smoldering myeloma compared to normal plasma cells (*Zhan et al*[60]; *P *= 0.0009) and in thyroid carcinomas compared to normal thyroid (*Huang et al*[61]; *P *= 0.005) (Figure 4). Interestingly, we found that *TACI *expression is associated with a poor prognosis in Burkitt lymphoma. TACI is significantly overexpressed in patients presenting Burkitt lymphoma who were dead at five years compared to patients alive at five years (Dave et al[62]; *P *= 0.004) (Figure 4). Recently, it was described that APRIL-TACI interactions mediate non-Hodgkin lymphoma B-cell proliferation through Akt, regulating cyclin D1 and P21 expression[63]. These data suggest that TACI is the main receptor for BAFF/APRIL in lymphoma cells and is associated with a bad prognosis. TACI therefore represents a potential lymphoma therapeutic target.

### Correlations between APRIL and heparan sulfate chain proteoglycans overexpression in tumor cells

Receptors for BAFF and APRIL are expressed exclusively by lymphoid cells. APRIL can bind and promote tumor-cell proliferation of several cell lines that do not express detectable mRNA for TACI and BCMA[9,64,65]. Ingold *et al *and Hendriks *at al *identified proteoglycans as the APRIL-non specific binding partners. This binding is mediated by HS side chains and can be inhibited by heparin[26,27]. Only APRIL expression was associated with a bad prognosis in several cancers. APRIL could sustain the survival of malignant cells directly through HS proteoglycans that can deliver signals through their intracellular tails upon binding to ligands[66,67].

Eleven HS proteoglycans has been identified so far – syndecan 1–4, glypican 1–6, CD44 isoforms containing the alternatively spliced exon v3 – and their gene expression can be evaluated by microarrays[68] (see Additional file 3). Interestingly, at least one HS proteoglycan gene was significantly overexpressed in cancers presenting an overexpression of APRIL compared to their normal counterparts (see Additional file 1). A similar observation was made for cancers showing an association between *APRIL *expression and the evolution of the disease (see Additional file 1).

HS proteoglycans regulate growth factor signaling, cytoskeleton organization, cell adhesion and migration[69]. HS proteoglycans are implicated in solid tumor development[70,71]. Syndecan-1 is absolutely required for the development of mammary tumors driven by the transgenic expression of the proto-oncogene Wnt-1 in mice[72]. The important role of HS proteoglycans is attributed in part to its highly negative electric potential, making it able to bind numerous proteins and to function as a coreceptor. However as APRIL activates cells that do not express TACI or BCMA, a direct signaling by the HS proteoglycans core protein is possible as already shown [66]. It may explain the overexpression of APRIL in association with disease aggressiveness in solid tumors which do not express BCMA or TACI. Nevertheless, additional studies are required to understand the exact role of APRIL in solid tumors, the existence of a signaling linked to the interaction of APRIL with HS proteoglycans and the possible existence of a receptor specific to APRIL not yet identified.

## Conclusion

The analysis reported here demonstrates that *APRIL *mRNA is overexpressed in at least 8 cancers compared to their normal counterparts, and within a given tumor category, is associated with a bad prognosis. These observations emphasize the interest using BAFF/APRIL inhibitors in the treatment of patients with cancers. We recently completed a phase I-II clinical trial of Atacicept TACI-Fc inhibitor showing the feasibility and safety of this targeting in multiple myeloma (submitted data). Heparin could be also useful in blocking APRIL binding to surface HS proteoglycan. Of note, we found here always one HS proteoglycan gene overexpressed in tumors that also highly expressed APRIL. A recent study reported that a brief course of subcutaneous low molecular weight heparin favorably influences the survival in patients with advanced malignancy and deserves additional clinical evaluation[73]. Thus, the targeting of APRIL or its interaction with HS proteoglycans may be promising in a large number of cancers.

## Competing interests

The authors declare that they have no competing interests.

## Authors' contributions

JM designed research, performed the analyses and wrote the paper. JLV and JDV have been involved in drafting and reviewing of the manuscript. BK is the senior investigator who designed research and wrote the paper. All authors read and approved the final manuscript.

## Pre-publication history

The pre-publication history for this paper can be accessed here:

http://www.biomedcentral.com/1471-2407/9/83/prepub

## Supplementary Material

Additional file 1 Expression of *BAFF, APRIL, TACI, BAFF-R *and *heparan sulfate proteoglycans *in human tumor types to that of their normal tissue counterparts using publicly available gene expression data, including the Oncomine Cancer Microarray database. Click here for file

Additional file 2 Classification of cancers included in the analysis. Click here for file

Additional file 3 Gene expression profile of Syndecan 1–4 and glypican 1–6 was determined using Oncomine Cancer Microarray database in bladder carcinoma, esophagus adenocarcinoma, brain tumors, breast cancer, head and neck tumors, lymphoma, pancreatic adenocarcinoma and adult male germ cell tumors presenting an overexpression of APRIL or an association with a bad prognosis or tumor progression. Click here for file
