# Supplementary material for: APRIL is overexpressed in cancer: link with tumor progression
Source: BMC Cancer. 2009 Mar 16;9:83. doi: 10.1186/1471-2407-9-83 (PMC2662875; doi:10.1186/1471-2407-9-83)
Supplement: Additional file 1 — Expression of BAFF, APRIL, TACI, BAFF-R and heparan sulfate proteoglycans in human tumor types to that of their normal tissue counterparts using publicly available gene expression data, including the Oncomine Cancer Microarray database. [file 1471-2407-9-83-S1.doc]

|  |  | **Gene overxpression compared to normal tissue counterpart** | | | | | |  |  |
| --- | --- | --- | --- | --- | --- | --- | --- | --- | --- |
| Tissue | dataset | BAFF | APRIL | TACI | BCMA | BAFF-R | Syndecan 1-4 | Glypican 1-6 | CD44 isoforms containing the alternatively spliced exon v3 |
| Blood | 2 | No | No | No | No | No | No | No | No |
| Leukemia | 33 | No | No | No | No | No | No | No | No |
| Lymphoma | 16 | **Yes** | **Yes** | No | No | No | **Yes** | No | No |
| Myeloma | 8 | No | No | **Yes** | No | No | No | No | No |
| Adrenal | 2 | No | No | No | No | No | No | No | No |
| Bladder | 8 | No | **Yes** | No | No | No | **Yes** | **Yes** | No |
| Brain | 23 | **Yes** | **Yes** | No | No | No | **Yes** | **Yes** | **Yes** |
| Breast | 44 | **Yes** | No | No | No | No | **Yes** | No | **Yes** |
| Cervix | 1 | No | No | No | No | No | No | No | No |
| Chondrosarcoma | 1 | No | No | No | No | No | No | No | No |
| Colon | 12 | No | No | No | No | No | No | No | **Yes** |
| Endocrine | 1 | No | No | No | No | No | No | No | No |
| Endometrium | 4 | No | No | No | No | No | No | No | No |
| Esophagus | 4 | **Yes** | **Yes** | No | No | No | **Yes** | **Yes** | **Yes** |
| Gastric | 5 | No | No | No | No | No | No | No | No |
| Head & Neck | 5 | No | **Yes** | No | No | No | **Yes** | **Yes** | **Yes** |
| Liver | 4 | No | No | No | No | No | No | No | No |
| Lung | 16 | No | No | No | No | No | No | No | No |
| Melanoma | 10 | No | No | No | No | No | No | No | No |
| Mesothelioma | 3 | No | No | No | No | No | No | No | No |
| Muscle | 2 | No | No | No | No | No | No | No | No |
| Neuroblastoma | 2 | No | No | No | No | No | No | No | No |
| Oral | 1 | No | **Yes** | No | No | No | No | No | **Yes** |
| Ovarian | 14 | No | No | No | No | No | No | No | **Yes** |
| Pancreas | 6 | No | **Yes** | No | No | No | **Yes** | No | No |
| Parathyroid | 1 | No | No | No | No | No | No | No | No |
| Prostate | 20 | No | No | No | No | No | No | No | No |
| Rectum | 2 | No | No | No | No | No | No | No | No |
| Renal | 11 | **Yes** | No | No | No | No | No | No | **Yes** |
| Salivary Gland | 1 | No | No | No | No | No | No | No | No |
| Sarcoma | 11 | No | No | No | No | No | No | No | No |
| Seminoma | 1 | No | No | No | No | No | No | No | **Yes** |
| Skin | 1 | No | No | No | No | No | No | No | No |
| Testis | 1 | **Yes** | **Yes** | No | No | No | **Yes** | **Yes** | No |
| Thyroid | 1 | No | No | **Yes** | No | No | No | No | No |
| Uterus | 1 | No | No | No | No | No | No | No | No |

**Table 1**

Expression of *BAFF, APRIL, TACI, BAFF-R* and *heparan sulfate proteoglycans* in human tumor types to that of their normal tissue counterparts using publicly available gene expression data, including the Oncomine Cancer Microarray database.
