# Supplementary material for: APRIL is overexpressed in cancer: link with tumor progression
Source: BMC Cancer. 2009 Mar 16;9:83. doi: 10.1186/1471-2407-9-83 (PMC2662875; doi:10.1186/1471-2407-9-83)
Supplement: Additional file 2 — Classification of cancers included in the analysis. [file 1471-2407-9-83-S2.doc]

**Classification of cancers included in the analysis :**

Breast Cancer grades :

Grading refers to the appearance of the cancer cells under the microscope. The grade gives an idea of how quickly the cancer may develop.

Grade I : cancer cells resemble normal cells and are not growing rapidly.

Grade II : cancer cells have features between grades I and III.

Grade III : cancer cells look more abnormal and may grow or spread more aggressively.

Cervical carcioma staging :

- - Stage 0 - full-thickness involvement of the [epithelium](http://en.wikipedia.org/wiki/Epithelium) without invasion into the [stroma](http://en.wikipedia.org/wiki/Stroma_(animal_tissue)) ([carcinoma in situ](http://en.wikipedia.org/wiki/Carcinoma_in_situ))
- - Stage I - limited to the cervix
  - IA - diagnosed only by microscopy; no visible lesions
    - IA1 - stromal invasion less than 3 mm in depth and 7 mm or less in horizontal spread
    - IA2 - stromal invasion between 3 and 5 mm with horizontal spread of 7 mm or less
  - IB - visible lesion or a microscopic lesion with more than 5 mm of depth or horizontal spread of more than 7 mm
    - IB1 - visible lesion 4 cm or less in greatest dimension
    - IB2 - visible lesion more than 4 cm
- - Stage II - invades beyond cervix
  - IIA - without parametrial invasion, but involve upper 2/3 of vagina
  - IIB - with parametrial invasion
- - Stage III - extends to pelvic wall or lower third of the vagina
  - IIIA - involves lower third of vagina
  - IIIB - extends to pelvic wall and/or causes [hydronephrosis](http://en.wikipedia.org/wiki/Hydronephrosis) or non-functioning kidney
- - IVA - invades mucosa of bladder or rectum and/or extends beyond true pelvis

- IVB - distant [metastasis](http://en.wikipedia.org/wiki/Metastasis)

Glioma grades :

**WHO grading system for astrocytomas**

- WHO Grade 1 — e.g., [pilocytic astrocytoma](http://en.wikipedia.org/wiki/Pilocytic_astrocytoma)
- WHO Grade 2 — e.g., diffuse or low-grade astrocytoma
- WHO Grade 3 — e.g., [anaplastic](http://en.wikipedia.org/wiki/Anaplastic) ([malignant](http://en.wikipedia.org/wiki/Cancer)) astrocytoma
- WHO Grade 4 — [glioblastoma multiforme](http://en.wikipedia.org/wiki/Glioblastoma_multiforme) (most common glioma in adults)

The prognosis is the worst for grade 4 gliomas, with an average survival time of 12 months. Overall, few patients survive beyond 3 years[1].

1. Mamelak AN, Jacoby DB: **Targeted delivery of antitumoral therapy to glioma and other malignancies with synthetic chlorotoxin (TM-601)**. *Expert Opin Drug Deliv* 2007, **4**(2):175-186.
