# Supplementary material for: APRIL is overexpressed in cancer: link with tumor progression
Source: BMC Cancer. 2009 Mar 16;9:83. doi: 10.1186/1471-2407-9-83 (PMC2662875; doi:10.1186/1471-2407-9-83)
Supplement: Additional file 3 — Gene expression profile of Syndecan 1–4 and glypican 1–6 was determined using Oncomine Cancer Microarray database in bladder carcinoma, esophagus adenocarcinoma, brain tumors, breast cancer, head and neck tumors, lymphoma, pancreatic adenocarcinoma and adult male germ cell tumors presenting an overexpression of APRIL or an association with a bad prognosis or tumor progression. [file 1471-2407-9-83-S3.pdf]

	Bladder carcinoma	Esophagus adenocarcinoma	Brain tumors	Head and neck tumors	Lymphoma	Pancreatic adenocarcinoma	Adult male germ cell tumor	Breast cancer
**Syndecan-1**	**Overexpressed in two independent studies (P = 2E-18 and 2.3E-4)[1,2]**		**Overexpressed in two idependant studies : P = 2.6E-13 and 6.2E-5 [3,4]**			**P = 1.4E-5 [5]**	**P = 1.4E-5 [6]**	**Overexpressed in two idependant studies : P = 1.3E-7 and 6.2E-4 [7,8]**
**Syndecan-2**				**Overexpressed in two idependant studies : P = 1.8E-4 and 5.5E-4 [9,10]**			**Overexpressed in two idependant studies : P = 1.5E-4 and 5.3E-9 [6,11]**	
**Syndecan-3**		**P = 1.6E-6 [12]**	**Overexpressed in two idependant studies : P = 2.1E-6 and 8E-6 [4,13]**	**P = 1.5E-5 [9]**			**P = 3.7E-4 [6]**	
**Syndecan-4**	**P = 9.5E-5 [1]**				**Overexpressed in two idependant studies : P = 3.5E-9 and 2.5E-8 [14,15]**			
**Glypican-1**	**Overexpressed in two independent studies (P = 6.4E-4 and 2.5E-4)[1,2]**	**P = 8E-6 [12]**	**Overexpressed in two independent studies (P = 2E-8 and 1.4E-4) [4,16]**	**P = 3.1E-7 [17]**			**P = 9.4E-4 [17]**	
**Glypican-2**			**P = 6.6E-6 [4]**					
**Glypican-3**							**P = 4.8E-20 [6]**	
**Glypican-4**			**P = 1.2E-8 [4]**				**P = 4.6E-9 [6]**	
**Glypican-5**								
**Glypican-6**			**Overexpressed in two idependant studies : P = 1.9E-7 and 3.2E-13 [3,4]**				**P = 1.3E-10 [6]**	
**CD44**		**P = 1E-4 [12]**	**Overexpressed in five indépendant studies (P = 4.5E-23, 5E-8, 3.4E-6 and 1.5E-5)[8, 3, 19, 16, 4]**	**P = 4.9E-6 [9]**			**P = 7.9E-18 [6]**	**P = 0.002 [7]**

Supplementary data Table B

Gene expression profile of Syndecan 1-4 and glypican 1-6 was determined using Oncomine Cancer Microarray database in bladder carcinoma, esophagus adenocarcinoma, brain tumors, brest cancer, head and neck tumors, lymphoma, pancreatic adenocarcinoma and adult male germ cell tumors presenting an overexpression of APRIL or an association with a bad prognosis or tumor progression.

1. Dyrskjot L, Kruhoffer M, Thykjaer T, et al. Gene expression in the urinary bladder: a common carcinoma in situ gene expression signature exists disregarding histopathological classification. Cancer Res. 2004;64:4040-4048.
2. Sanchez-Carbayo M, Socci ND, Lozano J, Saint F, Cordon-Cardo C. Defining molecular profiles of poor outcome in patients with invasive bladder cancer using oligonucleotide microarrays. J Clin Oncol. 2006;24:778-789.
3. French PJ, Swagemakers SM, Nagel JH, et al. Gene expression profiles associated with treatment response in oligodendrogliomas. Cancer Res. 2005;65:11335-11344.
4. Sun L, Hui AM, Su Q, et al. Neuronal and glioma-derived stem cell factor induces angiogenesis within the brain. Cancer Cell. 2006;9:287-300.
5. Iacobuzio-Donahue CA, Maitra A, Olsen M, et al. Exploration of global gene expression patterns in pancreatic adenocarcinoma using cDNA microarrays. Am J Pathol. 2003;162:1151-1162.
6. Korkola JE, Houldsworth J, Chadalavada RS, et al. Down-regulation of stem cell genes, including those in a 200-kb gene cluster at 12p13.31, is associated with in vivo differentiation of human male germ cell tumors. Cancer Res. 2006;66:820-827.
7. Richardson AL, Wang ZC, De Nicolo A, et al. X chromosomal abnormalities in basal-like human breast cancer. Cancer Cell. 2006;9:121-132.
8. Sorlie T, Perou CM, Tibshirani R, et al. Gene expression patterns of breast carcinomas distinguish tumor subclasses with clinical implications. Proc Natl Acad Sci U S A. 2001;98:10869-10874.
9. Ginos MA, Page GP, Michalowicz BS, et al. Identification of a gene expression signature associated with recurrent disease in squamous cell carcinoma of the head and neck. Cancer Res. 2004;64:55-63.
10. Toruner GA, Ulger C, Alkan M, et al. Association between gene expression profile and tumor invasion in oral squamous cell carcinoma. Cancer Genet Cytogenet. 2004;154:27-35.
11. Sperger JM, Chen X, Draper JS, et al. Gene expression patterns in human embryonic stem cells and human pluripotent germ cell tumors. Proc Natl Acad Sci U S A. 2003;100:13350-13355.
12. Hao Y, Triadafilopoulos G, Sahbaie P, Young HS, Omary MB, Lowe AW. Gene expression profiling reveals stromal genes expressed in common between Barrett's esophagus and adenocarcinoma. Gastroenterology. 2006;131:925-933.
13. Bredel M, Bredel C, Juric D, et al. Functional network analysis reveals extended gliomagenesis pathway maps and three novel MYC-interacting genes in human gliomas. Cancer Res. 2005;65:8679-8689.
14. Alizadeh AA, Eisen MB, Davis RE, et al. Distinct types of diffuse large B-cell lymphoma identified by gene expression profiling. Nature. 2000;403:503-511.
15. Rosenwald A, Wright G, Chan WC, et al. The use of molecular profiling to predict survival after chemotherapy for diffuse large-B-cell lymphoma. N Engl J Med. 2002;346:1937-1947.
16. Shai R, Shi T, Kremen TJ, et al. Gene expression profiling identifies molecular subtypes of gliomas. Oncogene. 2003;22:4918-4923.
17. Chung CH, Parker JS, Karaca G, et al. Molecular classification of head and neck squamous cell carcinomas using patterns of gene expression. Cancer Cell. 2004;5:489-500.
18. Bredel M, Bredel C, Juric D, et al. Functional network analysis reveals extended gliomagenesis pathway maps and three novel MYC-interacting genes in human gliomas. Cancer Res. 2005;65:8679-8689.
19. Liang Y, Diehn M, Watson N, et al. Gene expression profiling reveals molecularly and clinically distinct subtypes of glioblastoma multiforme. Proc Natl Acad Sci U S A. 2005;102:5814-5819.
